# The impact of the big five personality variables on self-employment survival

**DOI:** 10.3389/fpsyg.2022.1022477

**Published:** 2022-10-20

**Authors:** Thierry Volery, Jochen Mattes

**Affiliations:** ^1^School of Management & Law, Zurich University of Applied Sciences, Winterthur, Switzerland; ^2^Swiss Institute for Small Business and Entrepreneurship, University of St. Gallen, St. Gallen, Switzerland

**Keywords:** big five – Personality, self-employment, employment survival, panel (longitudinal) data analysis, entrepreneurship

## Abstract

Based on large, representative Australian household panel, this study investigates to what extent the Big Five personality variables influence self-employment survival and differentiates between successful or unsuccessful exit. In addition, the influence of two moderating variables, tertiary education and the motivation to become self-employed, are considered. Contrary to expectations, we found no impact of the Big Fives variable on self-employment survival in general. In the case of unsuccessful exit, we found that entrepreneurs with a higher level of Conscientiousness tend to stay self-employed although they may not be satisfied with their job. Similarly, entrepreneurs with a tertiary education prolong unsuccessfully self-employment stints, particularly if they exhibit higher level of Emotional Stability. Necessity-driven entrepreneurs exit unsuccessful stints earlier, especially if they exhibit a lower level of conscientiousness.

## Introduction

The personality of entrepreneurs has received a lot of attention in entrepreneurship since the 1960s. Much of the early research in entrepreneurship consisted of a series of large-scale studies conducted in an effort to understand the personal traits and characteristics of the entrepreneur: these were mainly carried out by behavioural scientists from disciplines such as psychology and sociology (Landström et al., 2012). One of the most influential works in this respect is David McClelland’s study “The Achieving Society” (1961). In this pioneer work, he demonstrated the link between the need for achievement in society and economic development.

By the late 1980s, several narrative reviews of the literature contended that there was no consistent relationship between personality and entrepreneurship (Gartner, 1988) and this stream of research eventually came to be regarded as something of a dead end. More recently, the role of personality in entrepreneurship has seen a revival as several meta-analyses provided evidence for the predictive validity of personality traits in entrepreneurship research (Rauch and Frese, 2006; Zhao and Seibert, 2006).

Overall, these recent studies suggest that the common variance of traits contribute to entrepreneurial behaviour. A substantial body of research indicates that personality variables play an important role in developing theories of the entrepreneurship process, including such areas as career choice (e.g., Zhao and Seibert, 2006; Caliendo et al., 2014), entrepreneurial cognition and opportunity recognition (e.g., Ardichvili et al., 2003), new venture survival (e.g., Ciavarella et al., 2004), and career success (e.g., Zhao et al., 2010; Wille et al., 2013).

To advance the field, scholars have suggested a need to clarify the role of personality in the entrepreneurship process though more longitudinal research (Hisrich et al., 2007). As past research has tended to focus on the start-up phase, it has been difficult to evaluate whether the personality variables are a predisposing factor or are learned from the role itself. In addition, personality characteristics that predict start-up behaviour may not predict behaviour later on in the entrepreneurship process. Rauch and Frese (2007) remarked that broad taxonomies of personality traits such as the Big Five have been less frequently used in entrepreneurship and that general traits have a lower predictability than specific traits such as locus of control or risk propensity. The heterogeneity of previous findings for personality variables suggests the presence of moderating variables which should be integrated in future research (Rauch and Frese, 2007).

The objective of this study is to investigate the impact of the Big Five personality variables (*Extroversion*, *Emotional Stability*, *Openness to Experience*, *Agreeableness*, and *Conscientiousness*) on self-employment survival by drawing on 12 waves of the Household, Income and Labour Dynamics in Australia (HILDA) survey. We define entrepreneurship in terms of self-employment, and we will use the terms ‘entrepreneurs’ and ‘self-employed’ interchangeably.

This study contributes to the field of psychology in entrepreneurship in three ways. First, we adopt a longitudinal approach, drawing on data which provide a sufficient time horizon to track self-employment survival in a meaningful way. In doing so, we aim for a more permanent effect. By investigating entrepreneurial stints and establishing when and why some individuals quit self-employment while others survive, this study sheds some light on the conditions for a sustainable entrepreneurship process. Second, our study complements previous research which attempted to evaluate the impact of the Big Five on entrepreneurial survival (Ciavarella et al., 2004; Caliendo et al., 2014) in that we differentiate between successful and unsuccessful exits. Third, we recognize that situational contingencies may be important and that there may be more than one type of entrepreneur or entrepreneurial venture (Zhao and Seibert, 2006). These different types of entrepreneurship may involve different skills and processes that require different theoretical explanations. Accordingly, we explore the impact of two moderating variables: tertiary education and financial prosperity.

## Personality and entrepreneurship

Personality theory provides a valuable framework for understanding and hypothesizing associations between traits and experiences in various life domains, including vocational life (Hogan, 1991). In other words, what people do—their behaviour—is a function of the kind of people they are—their personalities. Hogan et al. (1996) showed that scores on well-developed measures of normal personality are stable over reasonably long periods of time and predict important occupational outcomes. As such, a central assumption of personality theory is that an individual possesses a predisposition to behave, think, and feel in a relatively consistent manner over time and across diverse situations. This relative cross-situational consistency is captured by the term “personality trait.”

Personality has been conceptualized from a range of theoretical perspectives. After several decades of research on devising a general taxonomy of personality traits, a general consensus emerged in the early 1990s around the ‘Big Five’ (Barrick and Mount, 1991) personality dimensions: *Extroversion*, *Emotional Stability*, *Openness to Experience*, *Agreeableness*, and *Conscientiousness*. A substantial body of research has shown that several of the Big Five personality dimensions are related to employee job performance (e.g., Barrick and Mount, 1991; Rothman and Coetzer, 2003) and to entrepreneurial intentions and performance (Zhao et al., 2010).

In this study, we examine the impact of the Big Five on entrepreneurship survival and we distinguish between successful and unsuccessful exit as dependent variables at the end of the entrepreneurship stint. Specifically, we use the entrepreneur’s job satisfaction in the last year of self-employment as a proxy for success. In doing so, we follow Wennberg and DeTienne’s (2014) call to account for performance in empirical model when conducting research on entrepreneurship exit. As the authors remarked, “Many studies of exit in entrepreneurship have used exit to approximate the ‘failure’ of a new firm.” (Wennberg and DeTienne, 2014; p: 9) There is a need for a more nuanced approach: in the eyes of many entrepreneurs, exit and failure are two distinct concepts (Headd, 2003). We posit that job satisfaction can provide a synthetic perception of the success of the self-employment exit, capturing the satisfaction derived from the work content, meaningfulness, and remuneration. This perspective is particularly relevant is in the context of new independent firms, which are often run by one or a few entrepreneurs, and where the destiny of the firm is intimately linked to that of its owner(s).

### The Big five personality variables

In recent years, there has been an increased interest about the potential effect of the Big Five on entrepreneurship. In a first meta-analysis, Zhao and Seibert (2006) found significant differences between entrepreneurs and managers on four personality dimensions with entrepreneurs showing higher scores of *Extroversion*, *Conscientiousness* and *Openness to Experience*, and a lower score of *Agreeableness*. In a subsequent meta-analysis, Zhao et al. (2010) found that four of the Big Five personality dimensions were positively associated with entrepreneurial intentions and performance, with *Agreeableness* failing to be associated with either.

Ciavarella et al. (2004) were the first to analyze the relationship between the Big Five and venture survival. They found that the entrepreneur’s *Conscientiousness* was positively related to long-term venture survival and that *Extroversion*, *Emotional Stability*, and *Agreeableness* had no impact on survival. Contrary to their expectations, they found a negative relationship between *Openness to Experience* and long-term survival. However, Ciavarella et al. (2004) study suffered from two methodological weaknesses: a biased sample (graduates of a Southeastern university in the United States) and a small sample size (111 respondents, with only 57 included in the survival analysis).

A recent study by Caliendo et al. (2014) investigated the impact of the Big Five on the decision to become and stay self-employed by drawing on a large, representative German household panel. They observed the expected influence for just one dimension: the higher individuals score in *Agreeableness*, the higher their exit probability, revealing that low levels of *Agreeableness* positively support entrepreneurial survival.

In this section, we briefly describe each of the Big Five dimensions. We also report on the results of previous empirical studies which have investigated the impact of the Big Five in organizational behaviour and we formulate a series of hypotheses on how these personality dimensions relate to survival in self-employment.

*Extroversion*. This dimension refers to the degree of sociability or withdrawal that a person tends to exhibit. Extroverts are typically assertive, dominant, energetic, active, talkative, and optimistic. Introverts prefer to spend more time alone and are characterized as reserved, quiet, and independent. *Extroversion* is characterized by positive evaluations of life in general and career in particular (Clark and Watson, 1991), and there is evidence of positive associations between *Extroversion* and indicators of intrinsic career success such as job satisfaction (McCrae and Costa, 1991). Extroverted individuals tend to be cheerful, sociable, and seek excitement and stimulation, thus enabling them to develop social networks more easily, which may result in stronger partnerships with suppliers and customers (Baker, 1994). Another trait of *Extroversion* is the assertiveness of the individual (Barrick and Mount, 1991), and *Extroversion* has been identified as a strong predictor of leadership (Judge et al., 2002).

Although these traits have been identified as important for managers, it is plausible that all parts of the factor—building networks, being assertive and seeking leadership— are positively related to entrepreneurship. Social networks play a central role during the start-up and development stage. Entrepreneurs get support, knowledge, and access to finance and distribution channels through their social networks. They are also linked to other entrepreneurs and organizations in their industry and their region, and these contacts can widen the availability of resources that sustain a new firm. Thus,

*Hypothesis 1*: The greater the entrepreneur’s *Extroversion*, the longer the survival in self-employment.

*Emotional stability*. This dimension is also frequently referred to its converse—neuroticism. Emotionally stable individuals are characterized as usually calm, even-tempered, relaxed and able to face stressful situations without becoming upset. Individuals with a low level of *Emotional Stability* tend to experience a number of negative emotions including anxiety, hostility, impulsiveness, and vulnerability (McCrae and Costa, 1991). Studies investigating the relationship between *Emotional Stability* and job satisfaction have consistently found a positive correlation (Judge et al., 1998). Emotionally stable individuals can manage day-to-day performance pressure, remain optimistic, and generally maintain positive working relationships with coworkers (Hurtz and Donovan, 2000).

Entrepreneurs must be able to deal with uncertainty and stress when they launch a business venture. A large proportion of start-ups close in the first few years after they are established and there is an extra pressure to succeed when entrepreneurs invest their own money in the venture. In addition, the work of entrepreneurs is characterized by high pace and fragmentation within a relatively unstructured environment where they have the primary responsibility for all aspects of the venture (Mueller et al., 2012). Individuals with a high level of *Emotional Stability* should be able to tolerate these stressful situations. Similarly, those who are confident and self-secure are expected to prevail in this environment, resulting in a higher self-employment survival. Thus,

*Hypothesis 2*: The greater the entrepreneur’s *Emotional Stability*, the longer the survival in self-employment.

*Agreeableness*. An agreeable person is fundamentally altruistic, sympathetic to others and eager to help them, and in return believes that others will be equally helpful (John and Srivastava, 1999). A high level of *Agreeableness* characterizes cooperative individuals and a preference for interpersonal relationships. Conversely, someone at the low end of the dimension can be described as self-centered and hard-bargaining. *Agreeableness* leads to interpersonal trust which enhances collaboration, mutual supportiveness and shared norms and values.

In the field of entrepreneurship, Cable and Shane (1997) viewed the ability to build trusting relationships as a key factor to secure capital and future support from venture capitalists. In addition, agreeable entrepreneurs are better positioned to build alliances with other firms. For example, cooperation though product development alliances is an increasingly popular strategy that experienced and well-connected entrepreneurs use to cope with competitive markets and pioneering technologies (Eisenhardt and Schoohoven, 1996). We posit that entrepreneurs must exhibit *Agreeableness* to develop quality relationships with co-founders, employees, investors, suppliers and customers. This should increase their survival in self-employment and lead to a positive assessment of their entrepreneurial stint when they exit the market. Thus,

*Hypothesis 3*: The greater the entrepreneur’s *Agreeableness*, the longer the survival in self-employment.

*Conscientiousness*. This dimension contains two components. The first component reflects dependability. Conscientious individuals are careful, thorough, responsible, and organized (Barrick and Mount, 1991). The second component underpins volitional variables, suggesting that conscientious individuals are strong-willed, determined, and persistent. *Conscientiousness* has been found to be a consistent predictor of job performance across occupations involving managing others and sales performance (Hurtz and Donovan, 2000).

McClelland (1961) was the first to propose that individuals with a high need for achievement would be prone to become entrepreneurs because they have personal control over outcomes and are rewarded according to their own efforts. Conscientious entrepreneurs are hardworking, achievement-oriented, and persevering (Ciavarella et al., 2004), and this increases their persistence in self-employment. In their meta-analysis, Zhao and Seibert (2006) reported that entrepreneurs have a higher level of *Conscientiousness* than managers. These arguments suggest that conscientious entrepreneurs will have a longer survival in self-employment. Thus,

*Hypothesis 4*: The greater the entrepreneur’s *Conscientiousness*, the longer the survival in self-employment.

*Openness to experience*. People scoring low on openness tend to be conventional in behaviour and conservative in outlook. They prefer the familiar to the novel, and their emotional responses are somewhat muted. People scoring high on openness tend to be imaginative, broad-minded, curious, and non-traditional (Costa et al., 1991). Open-minded people have strong tendencies to seek out unfamiliar situations that allow for greater access to new experiences and perspectives. They are willing to entertain novel ideas and unconventional values, and they experience both positive and negative emotions more keenly than do closed individuals (Rothman and Coetzer, 2003).

Creativity, innovation, and change are all at the core of recent definitions of entrepreneurship (Shane and Vankataraman, 2000). This process requires intelligence and curiosity to acquire new knowledge, to combine resources and to develop innovative strategies to address unmet market needs. According to Patel and Thatcher (2014), *Openness to Experience* enables accurate assessments of environmental needs and enhances the creativity that is necessary to solve everyday problems and develop effective reactions to problems associated with small businesses. Thus,

*Hypothesis 5*: The greater the entrepreneur’s *Openness to Experience*, the longer the survival in self-employment.

### Moderation by education and financial prosperity

Past research found substantial unexplained variation in effect sizes for *Emotional Stability*, *Extroversion*, and *Openness to Experience* (Zhao and Seibert, 2006). In addition, several scholars have noted that there may be more than one type of entrepreneur or entrepreneurial venture and that these different types of entrepreneurship may involve different skills and processes that require different theoretical explanations (Ciavarella et al., 2004; Millán et al., 2012). This suggests that situational contingencies are important and points to the existence of moderators of the personality–entrepreneurship relationship. We consider two contingencies which are likely to affect self-employment entry and survival: education attainment and financial prosperity.

*Tertiary education*. Over the past decades, education has been described as a central component of human capital. Human capital theory assumes that people attempt to receive a compensation for their investments in human capital (Becker, 1975). Conceptually, education is thought to be linked to entrepreneurial efficiency and successful firm growth (Ertuna and Gurel, 2011) as individuals try to maximize their economic benefits given their human capital. We posit that the completion of a tertiary education will further strengthen the personality traits which will lead to entrepreneurship survival: Entrepreneurs who have a tertiary degree tend to set-up high value-added businesses where the personality of the owner-manager is likely to play a prevalent role. PhD graduates are unlikely to launch ‘mum and pop stores’ for which the rules of success and survival are well-established; instead, these individuals may pursue high value-added activities by leveraging on their personality, knowledge, ability and network. Amongst the Big Five, we argue that tertiary education will primarily be a moderator for *Conscientiousness*, *Emotional Stability*, and *Openness to Experience*.

University-educated people generally have good employment prospects and therefore face high opportunity costs to become self-employed (Cassar, 2006). Accordingly, they will want to minimize mishaps when pursuing a business idea and are thus likely to plan the launch of their business venture carefully and in an organized fashion, drafting for example a business plan or asking for advice. Once they have entered entrepreneurship, they are likely to raise their expected income from alternative employment and thus have higher performance requirements to remain in business (Unger et al., 2011). These outcomes tend to reinforce the dependability and volitional dimensions of *Conscientiousness.* More generally, the completion of a tertiary degree requires a lot of motivation and focus (O'Connor and Paunonen, 2007). The sense of purpose, hard work, and achievement gained by entrepreneurs during university studies is likely to motivate them in the pursuit of their venture idea later in their life.

Higher education invariably necessitates taking numerous examinations, completing assignments with tight deadlines, and thus dealing with stressful situations (O'Connor and Paunonen, 2007). Individuals with a tertiary education are therefore likely to have a high level of *Emotional Stability*, enabling a self-confident and relaxed approach to challenges. Later in their life, these individuals are well-equipped to deal with the uncertainty and stress of a new business venture and to persist in their project. Conversely, neurotic individuals tend to be anxious, depressed, insecure, and fearful (Chamorro-Premuzic and Furnham, 2003). They are more likely to experience anxiety and stress, compromising their performance.

Higher education is also likely to enhance *Openness to Experience* through the promotion of universalism, self-direction, and stimulation values (Roccas et al., 2002). These values emphasize intellectual and emotional autonomy, acceptance and cultivation of diversity and pursuit of novelty and change. The completion of a tertiary degree essentially requires individuals to consider new ideas. Entrepreneurs with higher education may be better able to manage new learning essential to both academic achievement and new business ventures, which in turn might increase persistence in entrepreneurship.

Consequently, we posit that the completion of tertiary education will strengthen the impact of *Conscientiousness*, *Emotional Stability*, and *Openness to Experience* on self-employment survival. Thus,

*Hypothesis 6a*: The relationship between the entrepreneur’s *Conscientiousness* and survival in self-employment is stronger for entrepreneurs with tertiary education.

*Hypothesis 6b*: The relationship between the entrepreneur’s *Emotional Stability* and survival in self-employment is stronger for entrepreneurs with tertiary education.

*Hypothesis 6c*: The relationship between the entrepreneur’s *Openness to Experience* and survival in self-employment is stronger for entrepreneurs with tertiary education.

*Financial prosperity*. There is a long tradition in economics and entrepreneurship to examine the relationship between wealth and business creation. Many studies have documented the positive relationship that exists between personal assets and the propensity to start a business and have interpreted this result as evidence of the existence and importance of liquidity constraints (Evans and Jovanovic, 1989; Fairlie and Krashinsky, 2012). Wealth matters for business ventures survival too. Holtz-Eakin et al. (1994) for example showed that liquidity constraints exert a noticeable influence on the viability of entrepreneurial enterprises.

Financial prosperity is therefore an important contingency variable in entrepreneurship: individuals who are financially prosperous are more likely to become and to remain self-employed. Even in under-performing ventures, financial prosperity is likely to prolong self-employment stints while entrepreneurs hope for a turnaround or an unexpected bonanza. Several studies suggested that entrepreneurs at the helm of such ventures remain committed to their project and continue to invest in a struggling venture (Åstebro et al., 2007).

We posit that financial prosperity will strengthen the relationship between three of the Big Five (*Extroversion*, *Emotional Stability*, *Openness to Experience*) and employment survival. First, financial prosperity is likely to increase the positive evaluations of life of extroverts in general and of their careers as entrepreneurs in particular. Having sufficient funds at their disposal will further boost extroverts who are characterized as cheerful and sociable and encourage them to stay self-employed. Second, we posit that financial prosperity will strengthen the impact of *Emotional Stability* on the length of self-employment survival. Sufficient funds provide entrepreneurs with a breathing space, reducing stress and anxiety, increasing their job satisfaction, and in turn increasing their persistence in entrepreneurship. In a similar fashion, financial prosperity is likely to alleviate their immediate liquidity worries, allowing them to better focus on other day-to-day pressing issues. This may in turn reinforce *Emotional Stability* and also prolong self-employment. Third, we anticipate that financial prosperity will strengthen the relationship between the entrepreneur’s *Openness to Experience* and survival in self-employment. Having sufficient funds allows open-minded entrepreneurs to further acquire new knowledge, test new formulae and try unconventional methods to solve customers’ problems. This in turn increases innovation and the likelihood of success in entrepreneurship. Thus,

*Hypothesis 7a*: The positive relationship between the entrepreneur’s *Extroversion* and survival in self-employment is stronger for financially prosperous entrepreneurs.

*Hypothesis 7b*: The positive relationship between the entrepreneur’s *Emotional Stability* and survival in self-employment is stronger for financially prosperous entrepreneurs.

*Hypothesis 7c*: The positive relationship between the entrepreneur’s *Openness to Experience* and survival in self-employment is stronger for financial prosperous entrepreneurs.

## Materials and methods

### Sample

We used data from the first 12 waves of the Household, Income and Labour Dynamics in Australia (HILDA) survey. HILDA is a nationally representative longitudinal household survey initiated by the Australian government. Initiated in 2001, the first wave covered 19,914 individuals in 7,682 households. In wave 11 this sample was topped up with an additional 2,153 households and 5,477 individuals.

Our sample contains entrepreneurship stints that started between 2002 and 2012. Stints that were already running in 2002 are not considered: Their inclusion in the sample would lead to a systematic underrepresentation of short stints. This procedure indeed reduces the sample size but ensures a fair representation of all stint lengths. In this sense, our sample presents a left truncation problem (or delayed entry).

The sample includes all types of entrepreneurs regardless of whether they had incorporated their business or not. In other words, our definition of entrepreneurs includes both owner-managers who operate their own incorporated businesses and people who operate their own unincorporated business. Individuals can have multiple self-employment stints. In our analysis, we solely consider the first observed stint. Including all stints would lead to an over-representation of individuals with multiple short stints and could thus lead to a bias. A total of 182 double self-employment stints are therefore excluded. This yields a total of 1,621 stints which we considered in this study. To check for robustness, we also ran the analysis based on the second observed stint (for entrepreneurs who have multiple stints). The regression results are provided in Appendix 1. They are comparable to those obtained in the original analysis. Relying on the second, rather than the first observed stints means that less exits are observed, and that consequently less stints can be classified as un−/successful. This, at least partially, explains the weaker significance levels.

*Cohorts*. Entrepreneurs exit self-employment through different means. For example, successful entrepreneurs might exit through an initial public offering or a trade sale, while unsuccessful entrepreneurs might be forced to wind up their business or to file for bankruptcy. The decision to exit and the exit strategy thus depend on the entrepreneur’s success. To differentiate between the types of exit, we rely on the entrepreneur’s job satisfaction in the last year of self-employment. Entrepreneurs with an above-median job satisfaction are coded as successful exit, the remainder as unsuccessful. A similar approach was adopted by Bates (2005), who asked entrepreneurs to assess their success after exit. Following this definition, our sample contains 213 successful and 573 unsuccessful stints. A further 835 stints cannot be classified because they were still running in the last year under analysis.

### Context

We acknowledge that entrepreneurship does not take place in a vacuum. A mix of attitudes, resources, and infrastructure, which altogether form the entrepreneurial ecosystem, are needed to support entrepreneurial activity. As highlighted by Thukral (2022), Australia has a good entrepreneurial ecosystem. Specifically, Australia provides favourable framework conditions for start-ups and Australian’s perceptions, attitudes, and beliefs towards entrepreneurship are generally positive. Australia, therefore, can be characterized as an “enabling context” (Stephan, 2018) for entrepreneurship marked by relative resource affluence, predictability, ease of transactions, and high legitimacy for entrepreneurs.

### Variables and measures

#### Dependent variable

The dependent variable is the length of the entrepreneurial stints. It indicates the survival in self-employment. In some cases, only upper and lower bounds of the stint-length can be established. This occurs when the entrepreneur does not respond to the HILDA survey for a period that overlaps with the entrepreneurship exit. We distinguish between successful and unsuccessful entrepreneurship stint by measuring the entrepreneurs’ job satisfaction in the last year of self-employment. Job satisfaction was measured by the single item: “All things considered, how satisfied are you with your job?”

#### Personality variables

The Big Five were measured with a 36-item inventory derived from Saucier (1994) set of adjectives. Participants were asked to rate how well each of the adjectives describes them using a 7-point scale (1 = does not describe me at all, 7 = describes me very well). Following this, a principal component analysis was performed. Internal reliability was assessed using Cronbach’s alpha: *Agreeableness* (α = 0.78); *Conscientiousness* (α = 0.78); *Emotional Stability* (α = 0.83); *Extroversion* (α = 0.73) and *Openness to Experience* (α = 0.73).

The personal variables were measured in wave 5 and 9. When the individual’s personality is not assessed in wave 5 we used information from wave 9. Personality traits are generally assumed to be stable among working-age adults (Hogan, 1991; John and Srivastava, 1999), and the values we observed remained consistent across the waves. The mean differences of personality scores between the wave 5 and wave 9 lie between −0.20 (*Agreeableness*) and 0.27 (*Openness to Experience*). This suggests that the error introduced by combining the values of the two waves is small.

#### Moderating variables

Our model includes two moderators: tertiary education and financial prosperity. Tertiary education was measured as dummy variable (0 = no tertiary education, 1 = at least one tertiary degree) at the beginning of the entrepreneurship stint. Financial prosperity was measured with a single item: “Given your current needs and financial responsibilities, would you say that you and your family are…” The responses ‘prosperous’, ‘very comfortable’, and ‘reasonably comfortable’ were coded as financially prosperous. The responses ‘just getting along’, ‘poor’, and ‘very poor’ were coded as not financially prosperous. Because the prosperity can change over time, we considered only the last known value before leaving self-employment.

#### Control variables

The following control variables are considered: gender (0 = male; 1 = female), age and age squared/100 (scaling of age is done to obtain regression coefficients of larger magnitude), migration background (0 = Australian native, 1 = migrant), and incorporation of business (0 = no; 1 = yes). All these variables (excepted financial prosperity) are captured at the beginning of the entrepreneurial stint.

### Analysis approach

We analyzed the survival dynamics of self-employment and differentiate between successful or unsuccessful exits. This was achieved by using two Multiple Risk Survival Models. The first model analyzes the characteristics of successful stints whereas the second model analyses the characteristics of unsuccessful stints. In the model for successful stints, the observations of unsuccessful stints are right censored at the time of exit. This approach has been widely adopted in past studies on entrepreneurship survival (Ciavarella et al., 2004; Millán et al., 2012). The survival lengths were modeled using a Weibull distribution. This parametric model allows for the inclusion of covariates of the survival times and of interval censored data (i.e., stint lengths for which only lower and upper bounds can be established). Traditional semi-parametric approaches for survival analysis such as the Cox regression lack this capability in their standard form.

In our parametric model, the entrepreneurial stint lengths were assumed to come from the Weibull distribution with density function: $f\left(t\right)=k\lambda t^{k-1}e^{-\lambda t^{k}}$ with time *t,* shape parameter *k > 0* and scale parameter λ>0 . The shape parameter k indicates how the exit rate changes over time. For *k < 1,* the exit rate decreases with time, for k = 1 it stays constant and, for *k > 1*, it increases with time. The reciprocal of the scale parameter λ indicates the time interval until ~63.2% of entrepreneurs have ended their stint.

The shape parameter *k* was assumed to be unaffected by the covariates. The shape parameter λ was regressed as: $\lambda =\lambda_{0}+\vec{\beta \prime }\vec{x}$ where $\lambda_{0}\;\mathrm{and}\;\vec{\beta }$ are regression parameters. The covariates thus modify the length of survival but not the exit rate change.

Two-hundred and thirty-one individuals (~14%) did not take the personality test. To retain these individuals in the sample, the values were mean-imputed. The results prove robust under moderate alterations. The covariates age, age^2^/100, and the Big-Five personality measures where centered before running the analysis. The descriptive statistics show the unaltered values. To take HILDA’s complex sampling method into account, we added the relevant terms for clustering and stratification. With 96 stratas, the model has 118 degrees of freedom. Finally, the observations were weighted using the weights provided by HILDA.

## Results

Table 1 shows the means, variances, and correlations of covariates for the whole sample, the successful stints, and the unsuccessful stints. In addition to the (un-) successful stints, the whole sample also contains stints that were not classifiable because they were still running at the end of the observation period. Consequently, the mean ‘lower stint length bound’ for the whole sample (2.86 years) lies above the mean for successful stints (1.84 years) and unsuccessful stints (2.28 years).

**Table 1:** Descriptive statistics and correlation matrix.

		**Total stints (N = 1,621)**				**Unsuccessful stints (N = 573)**																									
		**Mean**	**Var**	**Mean**	**Var**	**1**		**2**		**3**		**4**		**5**		**6**		**7**		**8**		**9**		**10**		**11**		**12**		**Mean**	**Var**
1	Lower stint length bound	2.86	6.70	1.84	2.68			0.07		0.03		−0.01		0.00		−0.02		−0.01		−0.05		0.08		0.09	^*^	−0.03		−0.03		2.28	4.06
2	Female	0.41	0.24	0.49	0.25	0.10				0.04		0.10	^*^	−0.09	^*^	0.02		0.07		0.02		0.03		0.28	^***^	0.08		−0.02		0.38	0.24
3	Age	41.78	162.53	41.60	173.02	−0.01		0.11				0.04		−0.04		0.13	^**^	0.03		−0.15	^***^	0.17	^***^	0.04		0.04		0.00		39.87	125.12
4	Business incorporation	0.42	0.24	0.28	0.20	−0.02		−0.02		−0.01				0.06		0.07		0.15	^***^	−0.09	^*^	−0.01		−0.09		0.02		0.23	^***^	0.26	0.19
5	Migration background	0.28	0.20	0.40	0.24	−0.06		−0.06		0.12		0.01				0.05		0.07		0.03		0.00		−0.01		0.07		−0.01		0.45	0.25
6	Tertiary education	0.21	0.17	0.17	0.14	−0.07		−0.06		0.01	^**^	0.00		0.09				0.03		0.00		0.03		0.04		0.09	^*^	0.03		0.22	0.17
7	Financial prosperity	0.67	0.22	0.79	0.17	−0.05		−0.03		−0.03		0.19	^*^	0.07		0.08				0.06		−0.01		−0.05		0.11	^*^	−0.05		0.64	0.23
8	Extroversion	4.00	1.22	4.06	1.43	−0.03		−0.03		0.13		0.09	^*^	0.14	^*^	0.13		0.08				0.01		−0.01		0.03		0.01		4.00	1.22
9	Emotional stability	4.59	0.90	4.77	1.01	0.05		0.06		−0.01		−0.09		0.16		0.17		−0.01		0.10				0.05		−0.04		0.03		4.50	0.83
10	Agreeableness	3.98	0.69	4.18	0.70	0.09		0.09	^**^	0.13		0.13		−0.06		0.00		0.08		−0.08		−0.03				−0.07		−0.10		3.93	0.70
11	Conscientious-ness	4.19	0.93	4.28	0.94	0.02	^*^	0.02		0.19		0.00		−0.14		0.07		0.00		−0.08		−0.06		−0.14				−0.13	^**^	4.13	0.85
12	Openness to experience	3.84	1.00	3.87	1.23	−0.15		−0.15		0.02		−0.01	^***^	−0.08		0.03		0.05		0.03		0.03		−0.12		0.01				3.84	0.92
				**Successful stints (N = 213)**																											

* *p* < 0.05;

** *p* < 0.01;

*** *p* < 0.001.

The control variables’ means are very similar for the two types of exit. Interestingly, the share of females in both samples is relatively high: 38% (unsuccessful) and 49% (successful). One possible explanation is HILDA’s stratification of the sample. Entrepreneurs of unsuccessful stints show lower scores in all personality dimensions.

We tested for multicollinearity among the covariates (gender, age, incorporation of business, migration, tertiary education, financial prosperity) and the Big Five personality variables by computing the variance inflation factors (VIFs). They range from 1.03 to 1.13, and, for unsuccessful stints, they range from 1.05 to 1.19. These values lie well below the recommended threshold of 10 (Neter et al., 1985). Overall, the correlation matrix suggests that personality constructs used in this study are not correlated and clearly distinct, and that they can be included in the analysis of the self-employment phenomenon.

Table 2 summarizes the regression results of both survival analyses. The results for successful stints are shown on the left and the results for unsuccessful stints are shown on the right side of the table. The scale parameters exp. (−0.04) = 0.96 for successful stints and exp. (−0.07) = 0.93 for unsuccessful stints are both smaller than 1. The risk of exit thus decreases with time. The risk of exit decreased faster for unsuccessful entrepreneurs. Our model fits the data very well (successful stints χ^2^ = 323.36, *p* < 0.00; unsuccessful stints χ^2^ = 359.66, *p* < 0.00 with 118 degrees of freedom).

**Table 2:** Impact of the big five on self-employment survival stints (regression results).

	**Successful stints**			**Unsuccessful stints**		
	**Effect**		**SE**	**Effect**		**SE**
(Intercept)	3.61	^***^	0.33	2.35	^***^	0.26
Gender	−0.32	^***^	0.10	−0.09		0.06
Age	0.13	^***^	0.02	0.01		0.02
Age^2^/100	−0.13	^***^	0.02	0.01		0.02
Incorporation of business	0.15		0.10	−0.13	^*^	0.06
Migration background	−0.20	^*^	0.10	−0.25	^***^	0.10
Openness to experience	0.17		0.09	−0.09		0.05
Conscientiousness	−0.14		0.08	−0.05		0.05
Extroversion	0.20	^*^	0.08	0.09	^*^	0.06
Agreeableness	−0.37	^***^	0.12	0.00		0.07
Emotional stability	−0.09		0.06	−0.06		0.10
Tertiary education	0.06		0.11	0.10		0.08
x Openness to experience	−0.08		0.13	0.06		0.09
x Conscientiousness	0.24	^*^	0.11	−0.03		0.07
x Extroversion	−0.46	^***^	0.09	−0.17	^***^	0.05
x Agreeableness	0.02		0.14	0.09		0.08
x Emotional stability	0.11		0.11	0.12		0.08
Financial prosperity	0.09	^*^	0.10	0.29	^***^	0.06
x Openness to experience	0.25	^*^	0.10	0.10		0.06
x Conscientiousness	0.00		0.11	0.12		0.07
x Extroversion	−0.07		0.10	−0.12		0.08
x Agreeableness	0.24		0.14	0.10		0.08
x Emotional stability	0.12		0.11	0.24	^***^	0.07
(Log Scale)	−0.04		0.03	−0.07	^***^	0.02
χ^2^	323.36			359.66		
Degrees of freedom	118			118		
p	0.00			0.00		
N	1,621			1,621		

* *p < 0.05;*

** *p < 0.01;*

*** *p < 0.001.*

Successful stints tend to be longer (*M* = 3.61 years) than unsuccessful stints (*M* = 2.35 years). The control variables offer interesting insights. Gender and age significantly influence the length of successful stints, but not the length of unsuccessful stints. Male entrepreneurs and older entrepreneurs tend to have longer successful stints. The effect is strongest at young ages and then flattens out. Successful stints of 21-year-old are 0.18 years longer than the ones of 20 year olds. Between 64-and 65-year-old entrepreneurs, the difference is only 0.07 years. The incorporation of a business leads unsuccessful entrepreneurs to exit earlier. A migration background significantly decreases the length of self-employment stints, irrespective of the success perceived.

As shown in Table 2, we find support for the influence of *Extroversion* on the stint length of both, successful and unsuccessful entrepreneurs. Higher *Extroversion* leads entrepreneurs to remain longer in self-employment (successful +0.20, unsuccessful: +0.09). Hypothesis 1 is thus accepted. *Agreeableness* significantly shortens successful stints (−0.37 years), but has no significant effect on the length of unsuccessful stints. Hypothesis 3 is partially accepted. The remaining effects of personality are not significant. This leads to the rejection of hypotheses 2, 4 and 5.

Tertiary education does not directly influence the time of (un-)successful exit in general. Yet, it moderates the effect of *Conscientiousness* on the length of successful stints. Entrepreneurs with higher *Conscientiousness* and a tertiary education delay successful exits. Hypothesis 6a is therefore accepted. Additionally, tertiary education inverts the earlier description of *Extroversion* on stint lengths. University educated extroverts tend to opt for an earlier exit, rather than to extend their stint, compared with their peers without tertiary education. We found no moderating effect of tertiary education on the relationship between the entrepreneur’s *Emotional Stability* and self-employment survival, and on the relationship between the entrepreneur’s *Openness to Experience* and self-employment survival. Hypotheses 6b and 6c are therefore rejected.

Overall, financial prosperity prolongs self-employment stints. This effect is stronger for unsuccessful entrepreneurs: Being financially prosperous, they can postpone the exit of their business venture. Financial prosperity strengthens this effect for entrepreneurs with high *Emotional Stability*. In the case of successful stints, financial prosperity strengthens the impact of *Openness to Experience* on the stint length. Similarly, in the case of unsuccessful stints, financial prosperity strengthens the effect if *Emotional Stability* on self-employment survival. Therefore, Hypothesis 7b is accepted and Hypothesis 7c is partially accepted. We found no evidence for a moderation of financial prosperity on the relationship between the entrepreneur’s *Extroversion* and survival in self-employment. Therefore, Hypothesis 7a is rejected.

## Robustness tests

We performed three robustness checks. The results can be found in Appendix 1. First, when an entrepreneur has multiple stints, we chose the first observed stint to increase the number of classifiable stints (i.e., stints that ended within the observation period). To ensure this did not bias the results, we reran the calculations using the second observed stint (where there were multiple stints). The coefficients closely resemble the ones of the original model. For the successful stints, two exceptions exist: *Agreeableness* loses in significance—potentially because fewer stints are classifiable as un/successful; and financial prosperity x *Emotional Stability* becomes significant. The effect retains its sign but becomes much stronger.

Second, we reran the analyses using the concept of ‘personality profile’, which combines the Big-Five into one measure. This approach was pioneered in a study about adolescents with an entrepreneurial Big Five constellation, characterized by high *Extroversion*, *Conscientiousness*, and *Openness*, and low *Agreeableness* and *Neuroticism*, and who were more likely than others to search for opportunities and develop entrepreneurial skills (Schmitt-Rodermund, 2004). A recent stream of research showed that this personality profile is a particularly robust predictor for entrepreneurial behaviour (Obschonka et al., 2010, 2013). As shown in Appendix 1, the control variables are comparable to the original model; the comparison demonstrates that an entrepreneurial personality leads to significantly longer self-employment stints.

Finally, we ran a log-logistic-based survival analysis. This analysis allows the hazard function to be non-monotonic (i.e., the risk of exit can increase for a certain time, and then decrease again). The resulting scale parameters are smaller than 1 for both cases [successful: exp. (−0.20) = 0.82; unsuccessful: exp. (−0.31) = 0.73], which implies that the best-fitting hazard function is monotonically decreasing. The usage of a Weibull distribution is thus adequate. The resulting covariates are very similar to the original values. However, differences exist in the significance levels.

## Discussion

With respect to entrepreneurship self-employment survival in general, we found evidence for a significant impact of *Extroversion* and *Agreeableness*. Specifically, higher *Extroversion*, which characterizes people as outgoing, gregarious, optimistic, and sociable, has a positive influence on the length of entrepreneurship stints. Although there is little evidence thus far about the impact of *Extroversion* on entrepreneurship from the literature on the Big Five, our results are in line with Lee and Tsang (2001) who found that high performing entrepreneurs tend to be more extroverted. Such entrepreneurs have more frequent communication with their business contacts and tend to have a larger number of contacts or a greater breadth of communication. More generally, extroverted entrepreneurs and their business ventures have been described as ‘active’ and ‘outward-looking’, in contrast to ‘passive’ and ‘inward-looking’ (Malecki and Poehling, 1999). As the quantity of information and the complexity of running a new venture grows, a high degree of external awareness, global information monitoring, and a capacity to develop effective social networks will favour entrepreneurs of all stripes. These characteristics are the hallmark of extroverted people.

In addition, *Agreeableness* significantly shortens successful stints, but has no significant effect on the length of unsuccessful stints. Therefore, entrepreneurs who end a successful stint tend to exhibit a low score in *Agreeableness,* pointing to self-centered and hard-bargaining traits. This finding is consistent with Caliendo et al. (2014) study of personality characteristics on entrepreneurship survival. The authors observed an influence for just *Agreeableness*: the higher the entrepreneur’s *Agreeableness*, the higher their exit probability, and therefore shorter self-employment stints. Contrary to much of the literature on entrepreneurship which bears a distinctly positive valence, our finding suggests that entrepreneurs can be Janus-faced in that positive attributes, such as resilience, self-efficacy, and need for achievement may sometimes devolve naturally into ruthlessness (Miller, 2015). After all, highly successful entrepreneurs, such as Bill Gates, Steve Jobs, and Mark Zuckerberg have been variously portrayed to be ruthless in their dealings with competitors, partners, and employees alike. An example of this lack of empathy is Elon Musk’s reaction when Mary Beth Brown, his longtime assistant, asked for a pay rise. Confronted with this request, he said he wanted to see if he could do her job, and then fired her instead (Vance, 2015).

As suggested by the previous studies on liquidity constraints (Holtz-Eakin et al., 1994; Fairlie and Krashinsky, 2012), our results indicate that entrepreneurs who are financially prosperous prolong their self-employment stint no matter if the exit is considered successful or unsuccessful. In doing so, they can invest in their established and successful business venture to grow it further. But financial prosperity may have a downside: instead of culling a poor project, the availability of liquidity may lead to an escalation of commitment where the entrepreneur continues to invest in a struggling venture. Conversely, our results show that tertiary education has no impact on the length of self-employment stints in general.

Considering education as a moderator, we observed that entrepreneurs with a tertiary degree prolong successful stints when they also have high scores in *Conscientiousness*. Tertiary education reinforces the effect of *Conscientiousness* and makes strong-willed, hard-working individuals persist in self-employment. The completion of a tertiary degree is likely to reinforce the dependability and volitional dimensions of *Conscientiousness*. The sense of purpose, hard work, and achievement gained by entrepreneurs during university studies is likely to motivate graduates in the pursuit of their venture idea later in their life. Conversely, tertiary education shortens self-employment stints of extroverted people.

The moderating effects of financial prosperity are in line with the theory on liquidity constraints (Fairlie and Krashinsky, 2012). Our findings suggest that financial prosperity strengthens the effect of *Openness to Experience* in prolonging successful entrepreneurship stints. Financial resources thus reinforce tendencies to seek out unfamiliar situations, to entertain novel ideas and try new ways to provide products and services. In other words, *Openness to Experience* allows entrepreneurs to engage in exploration activities through search, experimentation, and variation—all activities that increase the survival chance of a business venture (Volery et al., 2015). In addition, for unsuccessful stints, financial prosperity reinforces the effect of *Emotional Stability* on self-employment survival. Therefore, the availability of financial income and wealth further strengthen the tendency of unsuccessful entrepreneurs, who experience positive moods and emotions, to stick to a relatively unsuccessful project. Accordingly, for financially prosperous entrepreneurs, *Emotional Stability* may increase commitment and psychological inertia, causing them to postpone divestment for longer than rational reasoning would advise them to do (Sandri et al., 2010).

## Conclusion

The present study contributes to the vast stream of research on the personality of entrepreneurs. More specifically, we investigated the influence of the Big Five personality variables on entrepreneurial survival by drawing on a unique, representative dataset, the Household, Income and Labour Dynamics in Australia (HILDA) survey.

Our study makes two main contributions to the entrepreneurship and psychology literature. First, there have been heated debates about the role of personality in entrepreneurship (Gartner, 1989; Rauch and Frese, 2006; Zhao and Seibert, 2006). The current study extends our understanding of the role of personality in the entrepreneurship process. With respect to self-employment survival in general, we found evidence for a significant impact of *Extroversion* and *Agreeableness*. In a similar study, Patel and Thatcher (2014) found that greater *Openness to Experience* had a positive effect on self-employment survival, whereas individuals lower on *Emotional Stability* were less likely to persist in self-employment. For their part, Ciavarella et al. (2004) found that only *Conscientiousness* was positively related to long-term venture survival, and that there was a negative relationship between the entrepreneur’s *Openness to Experience* and long-term venture survival. Overall, our results, together with past research, suggest that the impact of the Big Five on self-employment survival is limited and, at best, inconclusive. While the personality structure of entrepreneurs is often distinctive compared to that of managers (Zhao and Seibert, 2006) and personality can play an important role in entrepreneurship entry (Zhao and Seibert, 2006; Caliendo et al., 2014), there exists a wide range of other factors which influence self-employment. These factors are most likely to be inherent to the business venture (e.g., profitability, growth, dynamic capabilities) and the industry (e.g., level of competition, environmental munificence), rather than to the personality of the entrepreneur.

Second, this study considered the role of tertiary education and financial prosperity as moderators to mitigate some of the heterogeneity identified in previous studies. In addition, we differentiated between successful and unsuccessful stints to account for performance at the time of exit. As suggested by the literature on liquidity constraints (Holtz-Eakin et al., 1994; Fairlie and Krashinsky, 2012), our results indicate that financially prosperous entrepreneurs prolong their self-employment stints regardless of whether the exit is considered successful or unsuccessful. Considering moderating effects, financial prosperity strengthens the effect of *Openness to Experience* in prolonging successful entrepreneurship stints. Recent research highlighted that openness personality factor may be the most important to predict entrepreneurship entry (Antoncic et al., 2015) and subsequent venture growth (Auer Antoncic et al., 2018). Our findings, suggest that this personality trait might be an important antecedent not only for starting a new business venture, but to remain involved with the business when the personal financial situation of the entrepreneur is stable. For unsuccessful stints, financial prosperity reinforces the effect of *Emotional Stability* on self-employment survival.

The findings suggest that tertiary education has no direct impact on self-employed survival. In line with human capital theory, we would have expected that entrepreneurs with tertiary education shorten their self-employment stint if it is unsuccessful. Given their qualification, they would expect to get a well-paid job as an employee, which raises their opportunity cost. If the venture was not performing according to their expectations, it was anticipated that they would exit quickly as they try to maximize their economic benefits given their human capital (Becker, 1975). However, these predictions did not materialize. Similarly, the moderating impact of tertiary education is limited: Tertiary education prolongs successful stints of conscientious entrepreneurs, and, conversely, it shortens both successful and unsuccessful self-employment stints of extroverted entrepreneurs.

The research also has some limitations. First, we base our analysis solely on data from Australia. Its history and similarity in culture suggests that the findings may also apply to European and North American contexts, but its validity in other regions of the world is unclear. Second, 835 stints could not be classified because they were still running in the last year under analysis. Third, entrepreneurs were asked to state how satisfied they were with their job at the end of their self-employment stint. In some circumstances, participants might have changed their opinion on their job during their stint. We could not take this change into account. Other measures of success should be considered in future studies. For example, entrepreneurs could be asked to reflect on their overall entrepreneurial experience, or whether they reached their personal and business goals. Finally, the scope of the personality variables considered in the present study was limited to the Big Five traits. It would be of interest to include other personality characteristics matching entrepreneurial tasks such as locus of control, need for achievement, and risk-taking propensity.

In conclusion, while the Big Five personality traits have been shown to influence entrepreneurship entry in past research, they play a relatively minor role in exit decisions and entrepreneurship survival in general. A fruitful avenue for future studies on survival could be to consider firm-level variables and to examine the interplay between personality and organization. Variables at the interface between these two levels, such as job satisfaction, job demand control, social support, and work-life balance could complement research on personality and bridge the gap with other streams of research in the entrepreneurship and organizational behaviour disciplines.

## Data availability statement

Publicly available datasets were analyzed in this study. This data can be found at: https://www.dss.gov.au/about-the-department/longitudinal-studies/living-in-australia-hilda-household-income-and-labour-dynamics-in-australia-overview.

## Author contributions

The authors conducted the statistical analysis and wrote this manuscript in its entirety as a team. All authors contributed to the article and approved the submitted version.

## Funding

Open access funding provided by Zurich University of Applied Sciences (ZHAW).

## Conflict of interest

The authors declare that the research was conducted in the absence of any commercial or financial relationships that could be construed as a potential conflict of interest.

## Publisher’s note

All claims expressed in this article are solely those of the authors and do not necessarily represent those of their affiliated organizations, or those of the publisher, the editors and the reviewers. Any product that may be evaluated in this article, or claim that may be made by its manufacturer, is not guaranteed or endorsed by the publisher.

## Author disclaimer

This paper uses unit record data from the Household, Income and Labour Dynamics in Australia (HILDA) Survey. The HILDA Project was initiated and is funded by the Australian Government Department of Social Services (DSS) and is managed by the Melbourne Institute of Applied Economic and Social Research (Melbourne Institute). The findings and views reported in this paper, however, are those of the author and should not be attributed to either DSS or the Melbourne Institute.

## Figures and Tables

**Table:** 

	**Second stint**				**Entrepreneurial personality**			
	**Successful stints**		**Unsuccessful stints**		**Successful stints**		**Unsuccessful stints**	
	**Effect**	**SE**	**Effect**	**SE**	**Effect**	**SE**	**Effect**	**SE**
(Intercept)	3.67***	0.67	2.01***	0.24	3.55***	0.35	2.36***	0.28
Gender	−0.29***	0.09	−0.02	0.06	−0.43***	0.09	−0.05	0.07
Age	0.13***	0.02	0.02	0.02	0.12***	0.02	0.01	0.02
Age^2^/100	−0.13***	0.03	0.00	0.02	−0.12***	0.02	0.01	0.02
Incorporation of business	0.19	0.10	−0.10	0.07	0.11	0.10	−0.15*	0.06
Migration background	−0.27**	0.10	−0.25***	0.07	−0.21	0.10	−0.25***	0.07
Openness to experience	0.14	0.09	−0.02	0.05				
Conscientiousness	−0.15	0.09	−0.05	0.05				
Extroversion	0.16*	0.08	0.10*	0.04				
Agreeableness	−0.23*	0.12	−0.03	0.07				
Emotional stability	−0.14	0.11	−0.10	0.06				
Tertiary education	0.05	0.12	0.12	0.08	0.11	0.10	0.10	0.07
x Openness to experience	0.06	0.16	0.00	0.09				
x Conscientiousness	0.26*	0.11	0.01	0.07				
x Extroversion	−0.41***	0.10	−0.02	0.06				
x Agreeableness	−0.09	0.15	0.13	0.08				
x Emotional Stability	−0.01	0.11	0.09	0.10				
Financial prosperity	0.04*	0.11	0.35***	0.06	0.02	0.10	0.27***	0.06
x Openness to experience	0.26*	0.11	0.06	0.07				
x Conscientiousness	−0.04	0.11	0.11	0.07				
x Extroversion	−0.11	0.10	−0.19**	0.06				
x Agreeableness	0.12	0.14	0.06	0.08				
x Emotional stability	0.26*	0.11	0.26***	0.07				
Entrepreneurial personality					0.08*	0.04	0.01*	0.03
x financial prosperity					0.06	0.05	0.02	0.03
x tertiary education					−0.09	0.05	0.03	0.03
								
(Log Scale)	−0.04	0.04	−0.07***	0.02	−0.03	0.03	−0.06***	0.02
χ^2^	322.83		351.65		279.06		324.44	
Degrees of freedom	119		119		106		106	
p	0.00		0.00		0.00		0.00	
N	1621		1621		1621		1621	

## Appendix 1

**p* < 0.05; ***p* < 0.01; ****p* < 0.001.
